# Survival Comparison between Melanoma Patients Treated with Patient-Specific Dendritic Cell Vaccines and Other Immunotherapies Based on Extent of Disease at the Time of Treatment

**DOI:** 10.3390/biomedicines7040080

**Published:** 2019-10-11

**Authors:** Robert Owen Dillman, Candace Hsieh

**Affiliations:** 1 Oncology Department, AIVITA Biomedical Inc., Irvine, CA 92612, USA; candace@aivitabiomedical.com; 2 Medical Oncology and Hematology Department, Hoag Cancer Institute, Newport Beach, CA 92663, USA; 3 Medical Oncology Department, University of California, Irvine, CA 92697, USA

**Keywords:** melanoma, dendritic cell, autologous tumor antigens, tumor initiating cells, vaccine, immunotherapy

## Abstract

Encouraging survival was observed in single arm and randomized phase 2 trials of patient-specific dendritic cell vaccines presenting autologous tumor antigens from autologous cancer cells that were derived from surgically resected metastases whose cells were self-renewing in vitro. Based on most advanced clinical stage and extent of tumor at the time of treatment, survival was best in patients classified as recurrent stage 3 without measurable disease. Next best was in stage 4 without measurable disease, and the worst survival was for measurable stage 4 disease. In this study, the survival of these patients was compared to the best contemporary controls that were gleaned from the clinical trial literature. The most comparable controls typically were from clinical trials testing other immunotherapy approaches. Even though contemporary controls typically had better prognostic features, median and/or long-term survival was consistently better in patients treated with this dendritic cell vaccine, except when compared to anti-programmed death molecule 1 (anti-PD-1). The clinical benefit of this patient-specific vaccine appears superior to a number of other immunotherapy approaches, but it is more complex to deliver than anti-PD-1 while equally effective. However, there is a strong rationale for combining such a product with anti-PD-1 in the treatment of patients with metastatic melanoma.

## 1. Introduction

The introduction of monoclonal antibody checkpoint inhibitors, especially the anti-programmed death molecule-1 (anti-PD-1) agents nivolumab and pembrolizumab, and anti-BRAF/MEK agents for patients with BRAF mutations, have revolutionized the treatment of metastatic melanoma. Anti-PD-1 agents have become the treatment of choice for the primary treatment of distant metastatic melanoma, and for the adjuvant treatment of high-risk surgically resected stage 3 and stage 4 melanoma, due to their curative potential [1]. However, there remains an unmet need because long-term disease control is still achieved in only a minority of patients. For this reason, there is a need for additional therapies, especially those that may be additive or synergistic with anti-PD-1 therapy without added toxicity [2,3,4]. In terms of mechanism of action, monoclonal antibodies to PD-1 and monoclonal antibodies to protein death molecule ligand (PDL-1) remove the enervating effects that result from the intercellular interaction of PD-1 and PDL-1 on cytotoxic T lymphocytes and other immune cells, thereby releasing suppressed immune responses that already existed in the host. In contrast, the mechanism of action of therapeutic vaccines is to induce new immune responses to tumor antigens, or to enhance weak existing immune responses to such antigens. For more than two decades, there has been great interest in the potential therapeutic application of dendritic cell vaccines (DCV) for patients with metastatic melanoma [5,6,7,8]. There are some commonly used approaches for generating dendritic cells from the peripheral blood and cryopreserving them [7,8,9,10], but there is tremendous variation in the sources of antigens for DCV [8,11,12,13]. Various investigators have consistently reported that such vaccines are well-tolerated and associated with desired antigen-specific immune responses, but rarely associated with significant clinical benefit [3,7,8].

Some of the most encouraging clinical results have been reported for a DCV consisting of autologous dendritic cells (DC) that were loaded with autologous tumor antigens (ATA) from autologous tumor cells that were self-renewing in tissue culture, and administered in granulocyte-macrophage colony stimulating factor (GM-CSF) [14,15,16,17,18]. Unlike most clinical investigations of DCV, the clinical trials with this DC-ATA vaccine have been associated with survival benefit. In a 54-patient single-arm phase 2 trial, the projected five-year survival was 54% at a time when median follow up was 4.5 years [16], and the eventual actual observed five-year survival was 50% with no patients lost to follow up. In a subsequent randomized phase 2 trial, the DC-ATA was superior to an irradiated autologous tumor cell vaccine that was also admixed with GM-CSF [17]. Long-term follow-up confirmed a doubling of median survival from 20.5 to 43.4 months, a higher observed survival rate at three years of 61% vs. 25%, and a 70% reduction in the risk of death [18].

Two of the major differences between these trials and most cancer vaccine trials is that the starting point for the preparation of the vaccine was surgical resection of tumor, and a short-term cell line had to be established as the source of ATA. Patients were typically referred for possible vaccine because they had surgically-resectable regionally recurrent stage 3 or distant stage 4 oligometastatic disease, or they were undergoing resection of a metastatic lesion for diagnostic or palliative reasons [19]. It was often several months later before the autologous cell line was available, and/or the patient was referred by their managing physician for treatment. As a result, in the interval from tumor collection to referral for treatment, many patients experienced recurrence and/or progression of disease, while others remained disease free, or they were rendered free of measurable metastatic disease by stereotactic radiation to the brain or resection of new metastases, or in rare cases by combination chemotherapy and biotherapy [19]. Survival varied depending on patients’ disease status at the time DC-ATA was initiated. The survival curves for each of these cohorts and their clinical characteristics were recently published [19]. Patients whose most advanced stage of melanoma was recurrent stage 3 and had no measurable disease at the time of DC-ATA treatment had a 72% survival rate at five years; patients whose most advanced stage of melanoma was stage 4, but had no measurable disease at the time of DC-ATA treatment had a 53% five-year survival; patients with measurable stage 4 disease had a median survival of 18.5 months and a two-year survival of 46% [19]. In the current study, efforts were made to identify comparable control groups from the literature for the purpose of survival comparisons between patients that were treated with DC-ATA and patients treated with other forms of immunotherapy.

## 2. Experimental Section

### 2.1. Melanoma Patients Treated with Patient-Specific Dendritic Cell Vaccines

The DCV-treated patients had metastatic melanoma and were enrolled in either a single-arm phase II trial (clinicaltrials.gov NCT00436930), or a randomized phase II trial (clinicaltrials.gov NCT00948480). Both clinical trials were conducted per the principles outlined in the Declaration of Helsinki and were approved by appropriate institutional review boards including the Hoag Hospital Institutional Review Committee for the Protection of Human Subjects (first approved 12 January 2000), the U.S. Food and Drug Administration (FDA; BBIND 8554), and reviewers for the National Cancer Institute’s Physician Data Query (clinical trial number: NCI-V01-1646) and the Western Institutional Review Board (Seattle, WA.) (first approved 01 February 2006, WIRB^®^ Protocol #20090753). All patients provided written informed consent prior to treatment. Details regarding these trials and the 72-DCV-treated patients were previously published [16,17,18,19].

### 2.2. Patient-Specific Dendritic Cell Vaccines

The manufacturing of the DCV-ATA product was previously described in detail [16,18,19]. Briefly, DC were derived from peripheral blood mononuclear cells that were obtained during a leukapheresis procedure and then cultured in interleukin-4 and GM-CSF. DC-ATA consisted of autologous DC that had been co-cultured with irradiated self-renewing autologous cancer cells for phagocytosis and loading of ATA. The cancer cells were grown in short-term cell cultures and they had characteristics and features of tumor initiating cells, including cancer stem cells and progenitor cells [11,20]. DC-ATA were suspended in GM-CSF at the time of subcutaneous injections intended for weeks 1, 2, 3, 8, 12, 16, 20, and 24.

### 2.3. Comparator Populations of Melanoma Patients Treated with Immunotherapy

The identification of comparable patient cohorts was achieved by a review of clinical trials in clinical trials.gov and articles that were identified in PubMed. An emphasis was placed on randomized trials that tested immunotherapy treatments. Preference was for studies conducted during 2000–2011, to coincide with the time when the patients of interest were treated with DCV.

### 2.4. Statistical Methods

There were no comparative statistical tests performed on the available data, because there were no analyses that were appropriate to perform.

## 3. Results

### 3.1. Stage 3 with No Measurable Disease

All of the stage 3 patients that were treated with DC-ATA had recurrent stage 3 disease, and many had recurred despite prior therapy. A comparable control group could not be identified from other trials, but some trials were limited to patients with stage 3 disease that had recently been eliminated by surgery. The comparative results for patients whose most advanced stage was 3 and had no measurable disease at the time of immunotherapy treatment, is shown in Table 1. One of the trials for comparison randomized 1,160 patients to either an allogeneic tumor cell vaccine + Bacillus Calmette-Guerin (BCG) that was called Canvaxin, or to BCG alone [21]. The majority of patients had primary microscopic stage 3, N1a and N2a microscopic disease involving small numbers of lymph nodes. In contrast, the DC-ATA-treated patients had recurrent, clinical stage 3, visible/palpable disease that was resected, N1b, N2b, N2c, or N3, which is associated with a worse prognosis [22]. The ECOG 1684 trial of interferon alpha was conducted in 280 patients who had primary stage 3 melanoma or recurrent stage 3 melanoma [23]; this trial was the basis for approval of that immune cytokine as the standard treatment of such patients in 1995. This trial was not limited to patients with stage 3 disease; it also included stage 2 patients with deep melanomas (>4 mm) and negative lymph nodes, in addition to patients with microscopic or clinically evident primary stage 3 melanoma, and recurrent stage 3 melanoma patients who had not received prior systemic treatment. Subsequent trials of adjuvant interferon included even higher proportions of patients with stage 2 disease and decreased proportions with stage 3 or recurrent stage 3 [24,25]; so, these trials were not included for comparison. Even though staging suggested that the comparator groups had a better prognosis, the rate of five-year survival was higher for the patients treated with DC-ATA. 

### 3.2. Stage 4 with No Measurable Disease

Table 2 shows the comparative results for patients whose most advanced stage was stage 4 but had no measurable disease at the time of immunotherapy treatment. In one report, data were pooled from five separate trials that were conducted at one institution [26]. These patients were comparable to the DC-ATA-treated patients in terms of the inclusion of patients who had been rendered surgically free of disease for stages M1a, M1b, and M1c, and both trials included some patients who had been treated for brain metastases, but all of the patients had HLA A-2 disease. Antigens in these vaccines included MAGE-3, MART-1, gp100, and tyrosinase. A few of these patients also received anti-CTLA-4. The other comparators were from arms of the Canvaxin trials for patients with resected stage 4 melanoma [21,27]. The design was similar to the trial for patients with resected stage 3 disease, in that the randomized trial tested the allogeneic tumor cell vaccine + BCG versus BCG. This trial was not restricted by HLA type. As shown in Table 2, even though the patients treated in the Canvaxin trial had less advanced disease, median survival was longer, and the percentage surviving more than five years was higher for the patients that were treated with DC-ATA. 

### 3.3. Stage 3 or 4 with No Measurable Disease

There was one large placebo-controlled randomized trial that explored the use of HLA-2 restricted peptides as a vaccine, with or without GM-CSF in patients who had been rendered free of disease by surgery [28]. One of the advantages of a patient-specific vaccine that utilizes autologous tumor antigens is that one does not have to rely on models that predict which HLA types will or will not recognize a specific antigen. An autologous vaccine is applicable to each individual patient, while an HLA-specific peptide vaccine is limited to patients of a specific HLA type. The results for these stage 3 and 4 patients were reported as a single pooled cohort. Table 3 shows the comparative results for patients whose most advanced stage was stage 3 or 4 and had no measurable disease at the time of immunotherapy treatment. The comparator study tested vaccines that included the HLA-2-restricted peptides MAGE-3, MART-1, gp100, and tyrosinase, which were given with or without GM-CSF; patients who were HLA-A2 negative were randomized to GM-CSF versus placebo [28]. Patients in the comparator arms had better prognostic features when compared to patients that were treated with DC-ATA [22]. About 60% of patients in the comparator studies had stage 3 disease, but this was mostly primary microscopic stage 3: N1a and N2a disease involving small numbers of lymph nodes. In contrast, among DC-ATA-treated patients, only 38% had stage 3, and all had recurrent clinical stage 3 disease rather than primary stage 3, and they all had visible &/or palpable N1b, N2b, N2c, or N3 disease. Furthermore, the majority of patients treated with DC-ATA had experienced stage 4 disease, while the majority of patients treated in the ECOG trial had stage 3 disease. Despite this, the rate of five-year survival was higher for patients that were treated with DC-ATA. 

### 3.4. Distant Stage 4 Measurable Disease

Table 4 shows the comparative results for patients whose most advanced stage was 4 and had measurable disease at the time of immunotherapy treatment. The survival data for interleukin-2 (IL-2) are from a randomized trial, in which all patients had to be healthy enough to receive IL-2, and were limited to patients who were HLA*A0201 positive, because the trial was testing the benefit of adding the gp100 peptide vaccine to IL-2 [29]. The survival data for anti-CTLA-4 comes from a report that pooled data from several trials conducted in previously treated patients [30]. The nivolumab anti-PD-1 data is from a phase II trial that tested the anti-PD-1 nivolumab in patients with metastatic melanoma who had previously been treated with one to five systemic treatments [31]. This patient population was similar to those treated with DC-ATA. There were no comparable data for pembrolizumab in a pretreated patient population.

### 3.5. Results for Immunotherapies Recently Approved for the Treatment of Melanoma

There were no survival comparisons among patients with minimal or no prior treatment for metastatic melanoma because DC-ATA was only administered to patients who had failed previous systemic therapies. However, in the past few years, numerous trials were conducted that tested anti-PD-1 in previously untreated or minimally treated melanoma patients, rather than heavily treated patients, and they have become the treatment of choice for first-line treatment. The results from these trials, as summarized in Table 5, demonstrate that, even in the first-line setting, objective tumor response and long-term survival are still not being experienced by most patients who have distant metastatic melanoma at the time of treatment. In the KEYNOTE-001 trial, the anti-PD-1 pembrolizumab was tested in 655 patients, 496 of whom had been previously treated (breakdown of numbers of patients who had 1, 2, or ≥ 3 prior treatments that are shown in Table 5) [32]. One article reported the response rates for all patients, regardless of whether they had measurable disease [32], while the other report focused on response only for patients who had measurable disease [33]. They reported results for the treatment naïve subset and for the whole group, but did not report specific data for the subset of patients who had been previously treated. However, based on the data provided in another analysis of this trial [33], one can determine that there were 134 objective responders among 448 previously untreated patients with measurable disease; accordingly, the response rate among previously treated patients was 134/448 = 29.9% (30%). Unfortunately, the specific progression-free and overall survival data for this subset was not included in any of these reports. In the CheckMate 066 trial, nivolumab was compared to chemotherapy [34]. In the KEYNOTE-006 trial, pembrolizumab was compared to the anti-CTLA-4 ipilimumab [35,36]. In the CheckMate 067 trial, nivolumab alone and nivolumab plus ipilimumab were both tested [37]. These studies excluded patients who had brain metastases, but patients with treated brain metastases were included in the DC-ATA trials.

The results for anti-PD-1 in patients with surgically resected stage 3 or stage 4 were not included in Table 1, Table 2 and Table 3 because of the limited long-term follow up data for such patients. However, there have been two large trials of adjuvant anti-PD-1 in high-risk melanoma. Table 6 summarizes the results from these trials. In the CheckMate 238 trial, nivolumab was compared to ipilimumab in patients with resected stage 3B, 3C, or stage 4 disease [37]. The other trial compared pembrolizumab to placebo in patients with resected stage 3A, 3B, or 3C [38]. Both trials were clearly positive with pretty similar one-year recurrence-free, or progression-free survival results. However, it should be noted that the patient population in the nivolumab trial was somewhat worse in that about 18% of patients had resected stage 4 disease, and the trial did not include stage 3A (clinically occult microscopic disease), while about 16% of patients in the pembrolizumab trial had stage 3A, and there were no patients with stage 4 disease. Furthermore, in both trials a significant proportion of patients with stage 3B disease only had microscopic metastases, while all of the stage 3 patients that were treated with DC-ATA had macroscopic metastases. Therefore, neither of these trials has a patient population comparable to those treated with the dendritic cell vaccine, and neither trial has reported out long term survival data.

## 4. Discussion

This study shows that the survival results for DC-ATA quite favorably compare with other immunotherapies that were available or being tested during 2000–2011. However, DC-ATA was clinically tested in melanoma patients in an era before the widespread availability of anti-PD-1 monoclonal antibodies or BRAF/MEK inhibitors. In fact, only one of the 72 DC-ATA-treated patients was ever treated with an anti-PD-1 during the five years of follow up. In more recent years, anti-PD-1 for metastatic melanoma patients [31,32,33,34,35,36], and anti-BRAF/MEK for those with BRAF mutations [39,40,41,42], have had a profound effect on the survival of patients with advanced melanoma. The checkpoint and BRAF/MEK inhibitors are immediately available for administration to patients, as they are “off-the-shelf” products, while DC-ATA requires surgical resection of a lesion, and even with current methods, it requires about eight weeks to manufacture the treatment product [20]. Furthermore, the anti-PD-1 and anti-BRAF/MEK products are associated with a high rapid objective response that is based on RECIST, while there were no objective responses in DC-ATA-treated patients per RECIST, although at least one patient did experience durable delayed complete regression of all the measurable sites of disease [18,43]. For these reasons, it does not appear that DC-ATA would displace any of these therapies in the treatment of stage 4 metastatic melanoma, but there is a strong rationale for combining DC-ATA with checkpoint inhibitors, especially in patients who lack an underlying anti-tumor immune response [3,4]. The mechanism of action of anti-PD-1 is the removal of immunosuppression of existing anti-tumor immune responses, while DC-ATA induces new anti-tumor immune responses or enhances the weak existing responses that are not immunosuppressed. Potential strategies for combining DC-ATA with checkpoint inhibitors include concurrent therapy with anti-CTLA-4 in patients who have progressed on anti-PD-1, or concurrent anti-PD-1 plus DC-ATA in patients who received anti-PD-1 alone while DC-ATA was being manufactured. In animal models, the best results were observed when anti-tumor vaccine was concurrently administered with anti-PD-1 therapy [44].

In addition to the checkpoint inhibitors, in recent years talimogene laherparepvec was approved for intralesional injections of metastatic melanoma [45]. Most of the tumor responses seen with this product appear to be the direct result of the cytolytic Herpes simplex virus in the construct, but it also contains GM-CSF that could help to promote a systemic immune response against the tumor antigens released by the cytolytic virus. The potential advantage of DC-ATA over such intralesional vaccines is that the latter are being injected into an immunosuppressive microenvironment that might inhibit antigen-loading of endogenous dendritic cells. With the DCV approach, antigens are only derived from self-renewing cancer cells rather than all cells in a tumor and they are loaded ex vivo away from such in vivo immunosuppression. The population treated with the intralesional cytolytic virus and GM-CSF was primarily patients with regional cutaneous metastases; so, there is no comparable cohort of DCV-treated patients with which to compare outcomes. Unfortunately, in the trial design that led to the approval of talimogene laherparepvec, the control arm was the same GM-CSF schedule of subcutaneous administration used in the ECOG 4697 trial rather than the more appropriate intralesional injection of GM-CSF as a control arm. The best results with talimogene laherparepvec were in patients with 3B, 3C, and M1a stage 4 disease (especially subcutaneous nodules). For this combination of measurable stage 3 and stage 4 patients, the objective response rate was 26%, the time to treatment failure was 8.2 months, median survival 23.3 months, and estimated two-year and three-year overall survivals were 50% and 39%, respectively. The rationale for combining DC-ATA with checkpoint inhibitors also applies to this cytolytic virus product, namely the potential immune recognition of additional ATA. Encouraging results have been reported for combining this agent with anti-CTLA-4 and anti-PD-1 agents [46,47]. Combining DCV with intralesional cytolytic virus injection may be additive or synergistic because of the direct anti-tumor effects of the cytolytic virus.

The major strength of this analysis is the focus on patient cohorts defined by stage and disease measurability at the time of treatment with immunotherapies. The most obvious weakness of this analysis is that it relies on comparisons to historical controls that are not perfectly matched, rather than direct comparison in randomized trials. However, an effort was made to match patients as closely as possible by stage and whether the disease was measurable at the time of treatment. Another weakness is the reliance on specific points such as median survival and two-year or five-year survival rates rather than a direct comparison of survival curves. Finally, the numbers of DC-ATA-treated patients in each of the subsets is rather small.

## 5. Conclusions

The survival outcomes for melanoma patients that were treated with DC-ATA vaccine compared favorably to other immunotherapies available or being tested at that time. In terms of convenience and rapidity of clinical benefit, this product does not appear to be as active as anti-PD-1 agents. There is a strong rational for combining DC-ATA vaccine with anti-PD-1 agents in concurrent or sequential treatment strategies due to the different mechanisms of action and toxicity.

## Figures and Tables

**Table 1:** Survival associated with immunotherapies for patients with stage 3 melanoma, but no measurable disease at the time of treatment.

Stage 3 Non-Measurable	DC-ATA Vaccine [19]	Allogeneic Tumor Cell Vaccine + BCG [21]	BCG [21]	Interferon Alpha [23]	Observation [23]
Median survival in months	> 60	> 60	>60	45.8	33.4
5-year survival	72%	59%	68%	46%	37%

DC-ATA = dendritic cell-autologous tumor antigens; OS = overall survival; BCG = Bacillus Calmette-Guerin.

**Table 2:** Survival associated with immunotherapies for patients with prior stage 4 melanoma, but no measurable disease at the time of treatment.

Stage 4 Non-Measurable	DC-ATA Vaccine [19]	Various Peptide Antigens [26]	Allogeneic Tumor Cell Vaccine + BCG [21,27]	BCG [21,27]
Median survival in months	> 60	46	35	39
5-year OS	53%	43%	42%	43%

DC-ATA = dendritic cell-autologous tumor antigens; OS = overall survival; BCG = Bacillus Calmette-Guerin.

**Table 3:** Survival associated with immunotherapies for patients with prior stage 3 or 4 melanoma, but no measurable disease at the time of treatment.

Stage 3 or 4 Non-Measurable	DC-ATA Vaccine [19]	Multiple Peptides [28]	GM-CSF [28]	Placebo [28]
Median OS	>60 mos	>60 mos	>60 mos	>60 mos
5- yr OS	60%	54%	52%	51%

OS = overall survival; GM-CSF = granulocyte macrophage colony stimulating factor; mos = months; DC-ATA = dendritic cell-autologous tumor antigens.

**Table 4:** Survival and response rates associated with immunotherapies in patients with measurable stage 4 metastatic melanoma at the time of treatment.

Stage 4 Measurable	DC-ATA Vaccine [19]	IL-2 [29]	IL-2 + GP-100 [29]	Anti-CTLA4 [30]	Anti-PD-1 [31]
Objective Response Rate	0%	10%	20%	≈12%	31%
Median survival in months	18.5	11.1	25.8	11.4	16.8
2-year survival	46%	18%	42%	22%	43%

IL-2 = interleukin-2; CTLA-4 = cytotoxic T lymphocyte antigen-4, PD-1 = programmed death protein-1.

**Table 5:** Trials of anti-PD-1 with or without anti-CTLA-4 in patients with metastatic melanoma who previously had or had not received systemic treatment.

Variables	Pembro [31,32]	Pembro vs. Ipi [34,35]	Nivo [33]	Nivo [36]	Nivo + Ipi [36]
Number of patients	655 Measurable disease = 581	556	210	316	314
Prior systemic therapies (*n*)	0 (159)	0–1	0	0	0
	1 (205)				
	2 (178)				
	≥ 3 (113)				
PFS	8.3 mos	8.4 mos	5.1 mos	6.9 mos (2-yr 37%)	11.5 mos (2-yr 43%)
OS	23.8 mos	32.7 mos	1-yr 70%	2-yr 59% 3-yr 52%	2-yr 64% 3-yr 58%
	2-yr 49%				
	5-yr 34%				
ORR	41% (267/655)	33%	40%	44%	58%
	33% (194/581)				
	0 prior therapies 45% (60/133)				
	>1 prior therapy 30% (134/438)				

Pembro = pembrolizumab; Nivo = nivolumab; Ipi = ipilimumab; PFS = progression free survival; OS = overall survival; ORR = objective response rate; mos = months; yr = year.

**Table 6:** Anti-PD-1 adjuvant therapy for resected high-risk melanoma.

Variables	Nivo vs. Ipi [37]	Pembro vs. Placebo [38]
Eligible	Resected stage 3 or 4 (3B, 3C, or 4 but not 3A)	Completely resected stage 3 including 3A
Number of patients	453 vs. 453	514 vs. 505
PFS	1-year 70.5% vs. 60.8%	1-year 75.4% vs. 61.0%
OS	Too early	Too early

PD-1 = programmed death protein-1; Ipi = ipilimumab; Pembro = pembrolizumab; PFS = progression-free survival; OS = overall survival.
